# Genetic diversity, SNP-trait associations and genomic selection accuracy in a west African collection of Kersting’s groundnut [*Macrotyloma geocarpum*(Harms) Maréchal & Baudet]

**DOI:** 10.1371/journal.pone.0234769

**Published:** 2020-06-30

**Authors:** Félicien Akohoue, Enoch Gbenato Achigan-Dako, Clay Sneller, Allen Van Deynze, Julia Sibiya

**Affiliations:** 1 Laboratory of Genetics, Horticulture and Seed Science, Faculty of Agronomic Sciences, University of Abomey-Calavi, Cotonou, Republic of Benin; 2 School of Agriculture, Earth and Environmental Sciences, University of KwaZulu-Natal, Pietermaritzburg, Republic of South Africa; 3 Biosciences Eastern and Central Africa (BecA) Hub, International Livestock Research Institute, Nairobi, Kenya; 4 Department of Plant Sciences, University of California, Davis, California, United States of America; University of Guelph, CANADA

## Abstract

Understanding the mechanisms governing complex traits variation is a requirement for efficient crop improvement. In this study, the molecular characterization, marker-trait associations and the possibility for genomic selection in a collection of 281 Kersting’s groundnut accessions were carried out. The diversity panel was phenotyped using an Alpha lattice design with two replicates in two contrasting environments. Accessions were genotyped using genotyping by sequencing technology. Genome-wide association analyses were performed between single nucleotide polymorphism markers and yield-related traits across tested environments. SNP markers were used to calculate the observed (Ho) and expected heterozygosity (He), and the total gene diversity (Ht). Genetic differentiation among accessions across ecological regions of origin was analysed. Our results revealed 493 quality SNPs of which 113 had a minor allele frequency>0.05, a total gene diversity of 0.43 and average Ho and He values of 0.04 and 0.22, respectively. Four clusters, highly differentiated by seed coat colour (Fst = 0.79), were identified. The population structure analysis showed two subpopulations with high differentiation across ecological regions (Fst = 0.37). The GWAS revealed 10 significant marker-trait associations, of which six SNPs were consistent across environments. The genomic selection through cross-validation showed moderate to high prediction accuracies for leaflet length, seed dimension traits, 100 seed weight, days to 50% flowering and days to maturity. This demonstrates the existence of genetic variability within Kersting’s groundnut and shows the potential for the improvement of the species. The findings also provide a first insight into the phenotype-to-genotype relationships in Kersting’s groundnut, using SNP markers.

## Introduction

With the challenges of global warming, farming land scarcity, and land demand for non-agricultural uses, the development of high yielding and climate-proof cultivars is one of the most relevant approaches to feeding the growing population [1, 2]. Currently, increasing the efficiency of breeding programmes requires the combination of conventional and molecular approaches for accurate selection and quick release of improved cultivars [3–5]. Developing molecular tools is the first step to applying enabling biotechnologies in cultivar development [5, 6]. Molecular markers help to dissect the variation of quantitative traits, such as yield, into the effects of quantitative trait loci (QTLs), and facilitate the transfer of those QTLs in new cultivars [7].

Recent advances in genomic technologies have mainly benefitted major crops species [8, 9], and the large diversity of other crops with great potential has received very little attention. Recently, the African Orphan Crop Consortium (AOCC) is sequencing the genome of 101 African crops to make data publicly available to accelerate breeding objectives. Meanwhile, there is an increase in food and nutritional insecurity, especially in developing countries [10]. Therefore, interventions to increase agricultural productivity and resilience to climate variations should emphasize crops species adapted to local agroecology [11, 12]. However, information on the diversity and the genetic systems governing traits of interest in such crops are still lacking, particularly for neglected grain legumes [6, 11, 12]. About ten neglected or orphan grain legume species were reported as nutritionally and economically important in tropical Africa [13] and these include Kersting’s groundnut [*Macrotyloma geocarpum* (Harms) Maréchal & Baudet].

Kersting’s groundnut originated from west Africa [14, 15]. The crop is grown by local populations in countries such as Benin, Ghana, Nigeria and Togo [15–18]. The crop was also reported in Central Africa especially in Cameroon [19, 20]. Kersting’s groundnut is cultivated for its grains that have high market value [15, 21]. In most west African countries, Kersting’s groundnut is preferred to cowpea [*Vigna unguiculata* (L.) Walp.] and bambara groundnut [*Vigna subterranea* (L.) Verdc.] due to the palatable taste of its grains [16, 19]. The grains of Kersting’s groundnut have a high nutritional value [22] and are considered as a healthy food especially for paediatric growth [18]. The dry grains of Kersting’s groundnut contain about 21.3% of crude protein and 6.2% of crude fibre [22, 23]. In addition, the grains are characterised by a high arginine and low-fat contents [24].

Despite its nutritional and economic importance, Kersting’s groundnut production is decreasing from year to year [15]. Major bottlenecks to the production of Kersting’s groundnut include the absence of high yielding, drought tolerant and disease resistant cultivars [15]. Unfortunately, the genetic diversity of Kersting’s groundnut has not been investigated to enable the implementation of relevant breeding programmes that will develop improved cultivars for farmers. Past studies on the genetic diversity of Kersting’s groundnut used only 19 enzymes on 20 accessions [20] to depict the variation in the crop. Pasquet *et al*. [20] reported a lack of genetic diversity among cultivated Kersting’s groundnut landraces. Biochemical markers on a small sample of Kersting’s groundnut may have revealed a narrow genetic base due to the low resolution provided by those markers [25, 26]. The renewed interest in orphan crops and the potential offered by the economical and nutritional values of Kersting’s groundnut call for actions towards creating high yielding and disease-resistant cultivars, with high nutritional value and adapted to drought prone environments.

Towards this effort, the development of highly informative DNA markers, including single nucleotide polymorphisms (SNPs), for a proper molecular characterization of Kersting’s groundnut germplasm [17] becomes crucial to speed up the selection process. SNP markers are abundant, highly polymorphic and informative to reveal with accuracy the existing diversity within crop species at the nucleotide level [5, 27, 28]. Moreover, the exploitation of the existing genetic diversity for cultivar development requires a clear understanding of the relationships between the genome and agronomic traits. Genome-wide association study (GWAS) is one of the popular genomic approaches to decipher genetic mechanisms controlling the variation of phenotypic traits. Among other advantages, the GWAS is a powerful tool offering a first insight into the genetic architecture of phenotypic traits variation [29–31]. Furthermore, the rapid and efficient selection of superior genotypes in Kersting’s groundnut breeding requires the development and the application of strong genomic selection (GS) and genomic-enabled prediction (GP) models. Unlike GWAS where markers are associated with traits of interest, GS is an integrated strategy exploiting molecular markers to advance breeding populations based on genetic estimated breeding values (GEBVs), which is particularly effective for complex traits like yield and flavour [32, 33]. Genomic selection accelerates the flow of candidate genes from genebank accessions to elite breeding lines, resulting in increased gains from selection [34].

Hence, the objectives of this study are to: (i) characterize the genetic diversity of Kersting’s groundnut using SNP markers, (ii) identify single nucleotide polymorphisms (SNPs) associated with morphological traits of interest in Kersting’s groundnut, and (iii) explore possibility for genomic selection in Kersting’s groundnut for accelerated cultivar development. We hypothesized that: (i) Kersting’s groundnut germplasm encompasses more genetic diversity, using SNP markers, contrary to Pasquet *et al*. [20] who reported an absence of genetic diversity within the species based on biochemical markers, (ii) polymorphic SNP markers are associated with traits of interest such as grain yield, flowering time, maturity time, number of seeds per plant, 100 seeds weight and number of pods per plant in Kersting’s groundnut, and (iii) cross-validation method revealed high genomic selection accuracies for key traits of interest in Kersting’s groundnut.

## Materials and methods

### Plant material

The material included 281 accessions of Kersting’s groundnut collected across Benin and Togo and held in the genebank of the Laboratory of Genetics, Horticulture and Seed Science (GBioS) of the University of Abomey-Calavi (UAC) in Benin. The diversity panel was collected from a wide range of agro-ecological regions, namely the Guinean, Sudano-Guinean and the Sudanian regions of Benin and Togo [15]. Accessions belonged to four landraces based on seed coat colour e.g. white seed coat (217), red seed coat (18), black seed coat (40) and white with black eye (6) (Table 1).

**Table 1:** Number of Kersting’s groundnut accessions per region and seed coat colour.

Landrace Region	White	Red	Black	White with black eye
**Guinean**	40	11	10	0
**Sudano-Guinean**	169	1	0	0
**Sudanian**	8	6	30	6

### Field trials and experimental design

The 281 accessions were phenotyped during the growth season of August 2017 to January 2018 at Sékou and Savè, two contrasting environments in Benin. Sékou is located in the Guinean phytogeographical zone characterized by an average rainfall of 1300 mm/year. Total rainfall during the growing season was estimated at 361 mm with an average temperature of 27.2°C. Savè belongs to the Sudano-Guinean zone characterized by an average rainfall of 1100 mm/year. In contrast to Sékou, the total rainfall recorded at Savè during the growing season was estimated at 161 mm from September to December 2017. The average temperature was estimated at 27.2°C. The experimental design was an alpha lattice design with two replications in each environment. This resulted in 562 experimental units for each trial. Each experimental unit was a ridge of 3.0 m long, containing 10 plants with 0.30 m inter-plant spacing [17, 35]. The field plan for the alpha lattice design was generated using R version 3.4.3 [36]. Kersting’s groundnut seeds were sown on 21^st^-22^nd^ August 2017 and the harvest was done from 3^rd^ to 6^th^ January 2018. Weeding was done systematically every two weeks in each location. Compound fertilizer NPK 15:15:15 was applied to plants four weeks after sowing at a rate of 100 kg/ha [37]. The Conti-Zeb 5_80% WP (mancozeb) fungicide was applied every two weeks with 500 g/ha to control fungal infestations.

### Field data collection

In total, 15 morphological traits were recorded during the field characterization (Table 2). Important traits evaluated were: diameter of the plant (DIP), plant height (PLH), leaflet length (LEL), leaflet width (LEW), petiole length (PEL), days to 50% flowering (DFF), and days to maturity (DTM). On a plant basis, the following were determined: grain yield per plant (GRY in g/plant), the number of seeds per plant (NSP), the number of pods per plant (NPP) and the number of seeds per pod (NSPod). Seed traits, namely seed length (SIL in mm), seed width (SWi in mm), seed thickness (STh in mm) and one hundred seeds weight (100SW in g) were collected (Table 2).

**Table 2:** Morphological traits and measurement techniques for phenotypic characterization of Kersting’s groundnut.

Traits	Code	Measurement techniques
**Diameter of plant (cm)**	DIP	Horizontal distance between two opposite points of the canopy
**Plant height (cm)**	PLH	Measured on 10 random plants from cotyledon scar to tip of plant
**Leaflet length (cm)**	LEL	Distance between the leaflet tip and the pulvinus measured on the third fully opened leaf from the tip
**Leaflet width (cm)**	LEW	Width of the broadest portion of the third fully opened leaf from tip measured
**Petiole length (cm)**	PEL	Measured on 10 plants from the base of petiole to beginning of limber
**Date to 50% flowering (days)**	DFF	Number of days from sowing when 50% of plants had at least one flower
**Days to maturity (days)**	DTM	Number of days from sowing that 50% of plant have mature pods
**Number of seeds per pod (seeds)**	NSPod	Count the number of seeds developed per pod on five plants randomly selected on each plot
**Number of pods per plant (pods)**	NPP	Count the number of pods developed per plant on five plants randomly selected on each plot
**Number of seeds per plant (seeds)**	NSP	Count the number of seeds developed per plant on five plants randomly selected on each plot
**Seed length (mm)**	SIL	Record on five seeds per plot. The seeds were chosen on five plants randomly selected on each plot
**Seed width (mm)**	SWi	Record on five seeds per plot. The seeds were chosen on five plants randomly selected on each plot
**Seed thickness (mm)**	STh	Record on five seeds per plot. The seeds were chosen on five plants randomly selected on each plot
**100 seed weight (g)**	100SW	Average weight of ten samples of 10 seeds at 10.5–11.5% moisture content. Estimated value is validated at a coefficient of variation below 5%
**Grain yield (g/plant)**	GRY	Weight of all seeds from each plant

### Phenotypic data analysis

Field data were explored for each trait for eventual outliers using the R package “outliers” [38]. For each trait, a mixed linear model was fitted per environment and across environments to estimate the best linear unbiased estimators (BLUEs) of accessions means using the META-R programme [39, 40]. The variation of morphological traits across environments was assessed through the construction of boxplots. We performed the analysis of variance (ANOVA) across environments, using BLUE-values and the R package “ggpubr” the function “*stat_compare_means()*” [41]. The ANOVA model was:

$$Y_{ijk}=\mu +G_{i}+E_{j}+{GE}_{ij}+R_{k}(E_{j})+\varepsilon_{ijk}$$ (1)

where the phenotypic response (Y_ijk_) is function of the overall mean (μ), the fixed effect of the i^th^ accessions (G_i_), the effect of the j^th^ environment (E_j_), the k^th^ replication (R_k_) within the j^th^ environment (E_j_), the genotype by environment interaction (GE_ij_) and the residual error (ε_ijk_).

To assess field heterogeneity, the coefficient of variation (CV) was calculated for each trait, using the formula:

$$CV=\frac{\sigma }{\mu }\;x\;100$$ (2)

where CV = coefficient of variation, μ = trait mean and *σ* = standard deviation

Furthermore, heritability estimates were obtained for each trait across environments using the META-R programme [40] to assess the feasibility of the GWAS. The formula for the broad sense heritability estimates was:

$$H^{2}=\frac{\sigma_{Acc}^{2}}{\sigma_{ACC}^{2}+\frac{1}{2}\sigma_{Acc:Env}^{2}+\frac{1}{4}\sigma_{Res}^{2}}$$ (3)

where $\sigma_{Acc}^{2}$ = variance of the accessions (Acc), $\sigma_{Acc:Env}^{2}$ = variance of the accession x environment (Env) interaction and $\sigma_{Res}^{2}$ = variance of the residual error.

The Pearson’s correlation matrix was also calculated between grain yield and other morphological traits using R version 3.4.3 [36] in order to select yield-related traits to include in the GWAS.

### DNA extraction and genotyping by sequencing

Kersting’s groundnut plants were grown at the University of Abomey-Calavi (Benin) under field conditions. Three-week old leaves were collected into 96 deep well samples collection plates and sent to the Integrated Genotyping Service and Support (IGSS) platform (https://ordering.igssafrica.org/cgibin/order/login.pl) located at Biosciences Eastern and Central Africa (BecA-ILRI) Hub in Nairobi for Genotyping. DNA extraction was done using Nucleomag Plant Genomic DNA extraction kit. The genomic DNA extracted was in the range of 50–100 ng/ul. DNA quality and quantity were checked on 0.8% agarose. Libraries were constructed according to Kilian *et al*. [42] Diversity Arrays Technology and Sequencing (DArTSeq) complexity reduction method through the digestion of genomic DNA and ligation of barcoded adapters followed by Polymorphic Chain Reactions (PCR) amplification of adapter-ligated fragments. Libraries were sequenced using Single Read sequencing runs for 77 bases. Next generation sequencing was carried out using Hiseq2500.

DArTseq markers scoring was achieved using the DArt Proprietary Limited (PL’S) proprietary SNP and SilicoDArt calling algorithms (DArTsoft14). SNP markers were scored as binary fashion for presence/absence (1 and 0, respectively) of the restriction fragment with the marker sequence in genomic representation of the sample. SNP markers were aligned to the reference genomes of mung bean [*Vigna radiata* (L.) R.Wilczek] and adzuki bean [*Vigna angularis* (Willd.) Ohwi & Ohashi] [43, 44], two related species of Kersting’s groundnut, in order to identify chromosome positions.

### Molecular analysis

We estimated minor allele frequency, observed (Ho) and expected heterozygosity (He), and total gene diversity (Ht) using the R package “adegenet” [45]. The total gene diversity (Ht), measured as the total expected heterozygosity, was calculated as follows: [46]

$$H_{t}=H_{s}+D_{st}$$ (4)

where H_t_ = total gene diversity of the total population as estimated from the pooled allele frequencies, H_s_ = within landrace diversity, D_st_ = between landraces diversity. Hs was estimated as follows:

$$Hs=1-\sum_{i=1}^{i=k}p^{2}$$ (5)

where p = frequency of the *i*^*th*^ allele at the *k*^*th*^ locus in each landrace and the value is averaged over all landraces. Likewise, Dst was calculated as:

$$Dst=(\sum_{i}\sum_{j}Dij)/s^{2}$$ (6)

where s = number of landraces, Dst = gene diversity between the *i*^*th*^ and *j*^*th*^ landrace. Dst was estimated as:

$$Dij=[\sum_{k}{\left(xik-xjk\right)}^{2}]/2$$ (7)

Where *x_ik_* = the frequency of the *k*^*th*^ allele in the *i*^*th*^ landrace, and *x_jk_* = the frequency of the *k*^*th*^ allele in the *j*^*th*^ landrace.

Missing marker data were imputed using the forest imputation method on the KDCompute sever (https://kdcompute.igss-africa.org/kdcompute/login), with the missForest algorithm based on multivariate unsupervised and supervised splitting techniques [47]. SNP markers with minor allele frequency (MAF) <0.05 were removed for the GWAS analysis.

### Clustering and population structure analysis

To assess the genetic diversity of Kersting’s groundnut accessions, the 493 SNP markers were used to calculate genetic dissimilarities among the 281 accessions including the four categories of landraces [48]. The genetic dissimilarities matrix was generating using marker data by calculating the presence/absence dissimilarity index with the “*Dice*” formula as follows:

$$d_{ij}=\frac{\left(b+c\right)}{2a+(b+c)}$$ (8)

with *dij* = dissimilarity between accessions i and j;

*a* = number of markers with xi = presence and xj = presence;
*b* = number of markers with xi = presence and xj = absence;
*c* = number of markers with xi = absence and xj = presence;
xi = SNP allele in the i^th^ accession, xj = SNP allele in the j^th^ accession.

The genetic dissimilarity matrix was used to generate an un-rooted tree using the weighted Neighbour-Joining (NJ) algorithm. Branches distances were used as criterion to weight the NJ tree, taking into account that errors in distances estimates are larger for longer distances [49]. Both the genetic dissimilarity matrix and NJ tree were determined in the Darwin software 6.0.4 [50]. To assess the genetic differentiation between pairs of clusters of Kersting’s groundnut accessions, a pairwise Fst analysis was performed using the R package “adegenet” [45]. Furthermore, the expected heterozygosity (He) was calculated using the function “poppr()” of the R package “poppr” to assess the level of genetic diversity within clusters of Kersting’s groundnut accessions [51]. Moreover, an analysis of variance (ANOVA) was conducted using all morphological traits to assess the phenotypic diversity among clusters, using the following model:

$$Y_{i}=\mu +C_{i}+\varepsilon_{i}$$ (9)

where the i^th^ phenotypic response (Y_i_) is a function of the overall mean (μ), the fixed effect of the i^th^ cluster (C_i_) and the residual error (ɛ_i_).

The population structure was also investigated using the Bayesian clustering method in STRUCTURE version 2.3.4 [52]. The three agro-ecological regions (e.g. Guinean, Sudano-Guinean and Sudanian regions) were included in the analysis as putative geographic origins of accessions. The length of the burn-in period and Markov Chain Monte Carlo (MCMC) were set at 10,000 iterations [53]. To obtain an accurate estimation of the number of populations, 20 runs were performed for each K-value (assumed number of subpopulations), ranging from 1 to 10. Further, Delta K-values were calculated and an appropriate K-value was estimated according to the Evanno *et al*. [53] method using STRUCTURE Harvester program [54]. At the appropriate K-value, Delta K-values make a salient break in slope of the distribution of likelihood values of K. Given a K-value, divergence rate of each subpopulation from a hypothetical ancestral population is estimated by population Fst values generated by STRUCTURE. The divergence rates show the extent of differentiation between subpopulations and the ancestral population for an accurate estimation of the clustering patterns. To complement the results of population structure, the pairwise Fst analysis was conducted among agro-ecological regions using the R software 3.4.3 [36] to check whether genetic differentiation among accessions was explained by their geographical origins. In addition, the two-sided Student test was performed on all morphological traits to compare means between both subpopulations.

### SNP-traits association analysis

The marker-trait association analysis was conducted per environment and across environments with heritability ≥0.50. Traits included grain yield per plant, days to 50% flowering, days to maturity, number of seeds per plant and number of pods per plant. The unified Mixed Linear Model (MLM) accounting for genetic relatedness (K-matrix) was used on BLUE-values estimated for each trait in order to control type I errors. The MLM analysis was conducted with and without including the three first principal components by using the GAPIT package of R software [55, 56]. The combination of different models is a good approach for the appropriate control of false positives and negatives in GWAS [57]. Therefore, only markers that revealed significant associations with both MLM and MLM-Q were retained as true phenotype-to-genotype associations [39]. The significant cut-off threshold was estimated using the Bonferroni correction threshold as follows: *p-value = 0*.*05/Me* with Me = the number of markers included in the analysis [39].

### Genomic prediction accuracy in Kersting’s groundnut

Genomic selection models were built for each morphological trait using the 493 SNP markers and the ridge regression analysis in the R package “rrBLUP” [33, 58]. The training and validation populations were defined through the stratified (all clusters) and “within cluster” sampling techniques [59, 60]. The stratified sampling technique refers to a random selection of accessions from each cluster in a way the training and validation populations consider the genetic diversity revealed by the cluster analysis within the crop [59]. In this study, about 75% of accessions were randomly selected from each cluster and included in the training population (211 accessions), while the remainder (70) formed the validation population. Contrary to the stratified sampling technique, the “within cluster” sampling technique consists in a random selection of accessions from one cluster to form both training and validation populations [59]. This sampling technique considers only the genetic diversity within one cluster of accessions for genomic prediction. Therefore, 162 accessions were randomly selected in cluster I (essentially composed of white seeded accessions) to form the training population while the rest of the accessions (55) of this cluster were used as the validation population. Correlation coefficients between observed and predicted values of all traits were calculated, using the cross validation approach to assess the accuracy of the genomic selection models.

## Results

### Morphological traits variation and association patterns in Kersting’s groundnut

**Fig 1:**
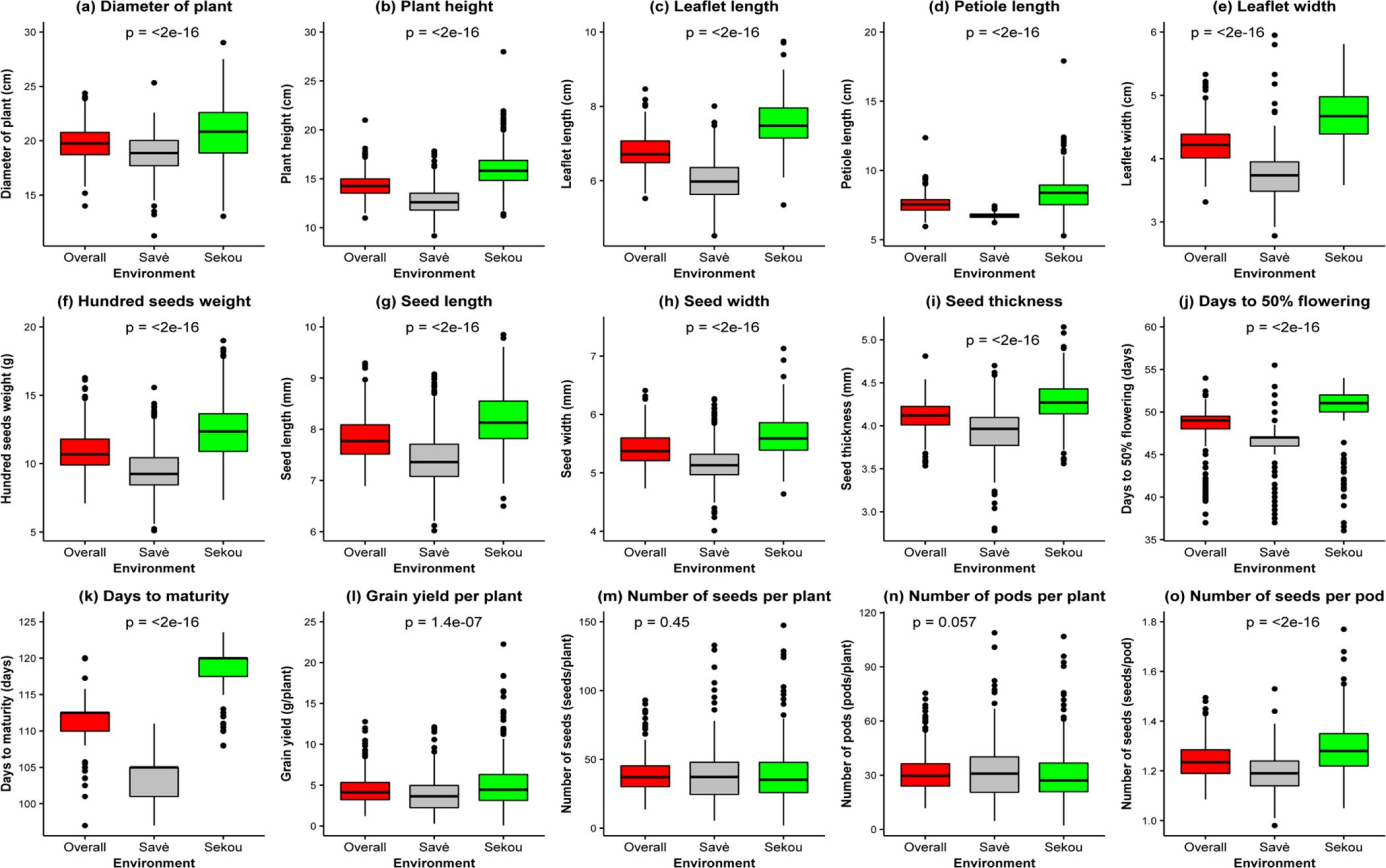
Variation of the 15 morphological traits across environments.

Highly significant (p<0.001) genetic variation was observed among accessions for all morphological traits, except seed thickness (Table 3). The genotype x environment (GxE) interaction was also highly significant for most traits except leaflet width, seed thickness, days to maturity and number of seeds per pod (Table 3). Average performances were lower at Savè than Sékou for all morphological traits (Fig 1). The coefficients of variation (CVs) and broad sense heritability estimates across environments for the 15 morphological traits are shown in Table 4. The coefficients of variation were <20% for most traits including the diameter of the plant, plant height, leaflet length, leaflet width, petiole length, 100 seeds weight, seed length, seed width, seed thickness, and the number of seeds per pod. In contrast, higher coefficients of variation were obtained for grain yield per plant (42.2%), the number of seeds per plant (36.3%) and the number of pods per plant (34.9%), revealing that there was a high variability for those traits across environments (Table 4). Moreover, the broad sense heritability estimates were high for 100 seeds weight (0.61), days to 50% flowering (0.86), days to maturity (0.87), grain yield per plant (0.53), number of seeds per plant (0.55) and number of seeds per pod (0.52) (Table 4).

**Table 3:** Mean squares of analysis of variance of morphological traits among Kersting’s groundnut accessions across Sékou and Savè environments.

Variables	Accession	Environment	Acc*Env	Residuals
	df = 280	df = 1	df = 270	df = 532
**DIP**	10.76***	1305.11***	10.51***	4.73
**PLH**	6.62***	2953.04***	5.19***	2.54
**LEL**	0.77***	734.42***	0.65***	0.64
**PEL**	4.90***	742.31***	4.00***	2.12
**LEW**	0.41*	245.862***	0.35ns	0.33
**100SW**	10.27***	2322.55***	4.92***	3.04
**SIL**	0.71***	140.68***	0.49*	0.40
**SWi**	0.33***	53.83***	0.21**	0.16
**STh**	1.40ns	22.10***	1.44ns	1.43
**DFF**	45.10***	4014.20***	7.90***	3.20
**DTM**	92.00***	55469.00***	48.00ns	49.00
**GRY**	14.39***	516.67***	15.85***	8.95
**NSP**	797.90***	281.7ns	888.80***	507.80
**NPP**	470.23***	924.12ns	522.87***	292.66
**NSPod**	0.02***	2.30***	0.01ns	0.01

DIP = diameter of plant (cm), PLH = plant height (cm), LEL = leaflet length (cm), LEW = leaflet width (cm), PEL = petiole length (cm), 100SW = 100 seed weight (g), SIL = seed length (mm), SWi = seed width (mm), STh = seed thickness (mm), DFF = days to 50% flowering (days), DTM = days to maturity (days), GRY = grain yield (g/plant), NSP = number of seeds per plant, NPP = number of pods per plant, NSPod = number of seeds per pod; df = degree of freedom, Acc = accession, Env = environment, ns = non-significant

*, **, *** indicate significance at p-values of 0.05, 0.01, and 0.001, respectively.

**Table 4:** Coefficients of variation and broad sense heritability estimates of Kersting’s groundnut morphological traits across Sekou and Savè environments.

Traits	Code	CV (%)	H
**Diameter of plant (cm)**	DIP	8.50	0.47
**Plant height (cm)**	PLH	8.98	0.51
**Leaflet length (cm)**	LEL	6.62	0.41
**Petiole length (cm)**	PEL	8.98	0.46
**Leaflet width (cm)**	LEW	7.57	0.29
**Hundred seeds weight (g)**	100SW	14.94	0.61
**Seed length (mm)**	SIL	5.62	0.36
**Seed width (mm)**	SWi	5.54	0.39
**Seed thickness (mm)**	STh	4.38	0.15
**Days to 50% flowering (days)**	DFF	7.06	0.86
**Days to maturity (days)**	DTM	3.62	0.87
**Grain yield per plant (g/plant)**	GRY	42.22	0.53
**Number of seeds per plant (seeds)**	NSP	36.26	0.55
**Number of pods per plant (pods)**	NPP	34.89	0.52
**Number of seeds per pod (seeds)**	NSPod	5.65	0.47

CV = coefficient of variation, H = broad sense heritability

Furthermore, the Pearson correlation analysis revealed highly significant (p<0.001) positive correlations of grain yield per plant with the yield components, 100 seed weight, number of seeds per plant, number of seeds per pod, and number of pods per plant at Sékou, Savè and across environments (Table 5). In addition, there were significant negative correlations between grain yield per plant, days to 50% flowering and days to maturity for all environments. Moreover, a significant positive correlation was detected between grain yield per plant and seed thickness at Savè (Table 5). GRY was poorly correlated to leaf morphological traits.

**Table 5:** Pearson correlations between grain yield per plant and other Kersting’s groundnut morphological traits.

Traits	Code	Sékou	Savè	Overall
**Diameter of plant (cm)**	DIP	0.04	0.10	0.02
**Plant height (cm)**	PLH	0.01	0.14*	0.08
**Leaflet length (cm)**	LEL	-0.01	0.13*	0.03
**Petiole length (cm)**	PEL	0.02	0.10	0.04
**Leaflet width (cm)**	LEW	0.03	0.09	0.04
**Hundred seeds weight (g)**	100SW	0.58**	0.46**	0.51**
**Seed length (mm)**	SIL	0.10	0.14*	0.24**
**Seed width (mm)**	SWi	0.12*	0.11	0.24**
**Seed thickness (mm)**	STh	-0.02	0.44**	0.09
**Days to 50% flowering (days)**	DFF	-0.42**	-0.03	-0.45**
**Days to maturity (days)**	DTM	-0.38*	-0.04	-0.40**
**Number of seeds per plant (seeds)**	NSP	0.96***	0.95***	0.93***
**Number of pods per plant (pods)**	NPP	0.93***	0.94***	0.88***
**Number of seeds per pod (seeds)**	NSPod	0.40***	0.21**	0.34***

*, **, *** indicate significance at p-values of 0.05, 0.01, and 0.001, respectively

### Single nucleotide polymorphisms in Kersting’s groundnut

In total, the high density Genotyping by Sequencing (GBS) of the 281 accessions yielded 493 single nucleotide polymorphisms (SNPs) with 0.3–30.9% of missing data. The call rate ranged from 63 to 100% with an average of 0.96±0.05. The reproducibility of markers ranged from 0.91 to 1.00 with an average of 0.99±0.02. Only 10.9% (54) of SNPs were aligned to the reference genomes of both adzuki bean and mung bean. The average minor allele frequencies frequency (MAF) was 0.04±0.07. About 22.9% (113) of markers had minor allele frequency greater than 0.05 (S1 Table). Moreover, mean observed and expected heterozygosity were estimated at 0.04±0.08 (0 to 0.64) and 0.22±0.09 (0.11 to 0.46) respectively. The total gene diversity (H_T_) across markers varied from 0.07 to 0.50; the average H_T_ value was 0.43 (S1 Table). Considering the low proportion of markers aligned to the reference genome of related species, both aligned and non-aligned SNP markers were considered for association analysis.

### Genetic diversity of Kersting’s groundnut germplasm

**Fig 2:**
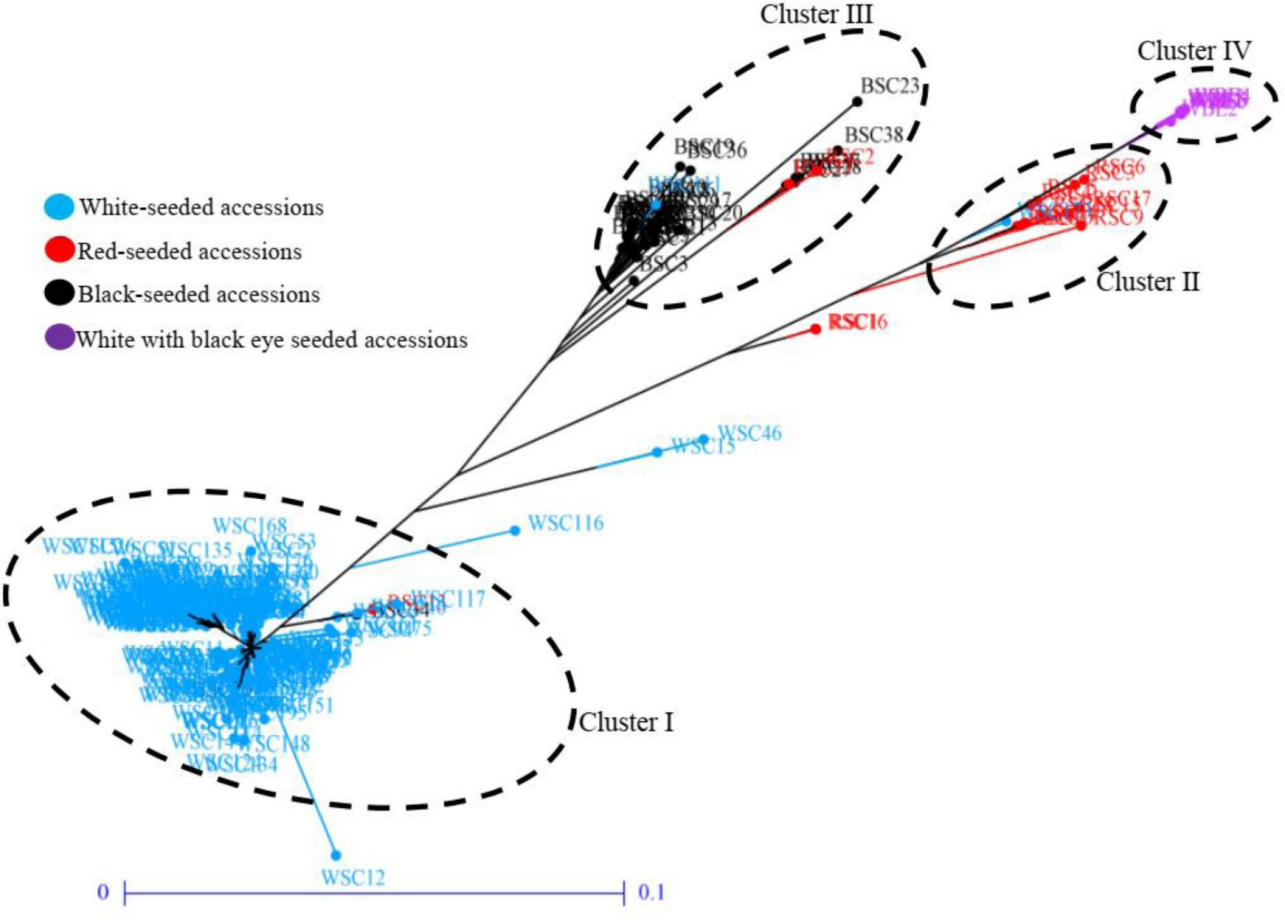
Un-rooted Neighbour-Joining (NJ) tree showing the relatedness among the 281 accessions of Kersting’s groundnut.

The clustering groups the 281 accessions into four clusters based on shared attributes (Fig 2). Cluster I (77.2% of accessions) was mainly composed of white seeded accessions, which were highly related to each other and clearly separated from other accessions. Cluster II (6.4% of accessions) was composed of red seeded accessions, which were highly related to each other. Cluster III (14.2% of accessions) was essentially composed of black seeded accessions. Moreover, cluster IV (2.1% of accessions) was exclusively composed of white with black eye accessions, revealing a high genetic relatedness among those accessions (Fig 2). The clustering was supported by results of the pairwise Fst analysis between pairs of clusters (Table 6). The overall Fst-value was 0.62, showing a high genetic differentiation among clusters of accessions. In addition, the pairwise Fst-values ranged from 0.30 to 0.92. The lowest Fst was obtained between Clusters II and IV while the highest Fst-value was revealed between Clusters I and IV (Table 6).

**Table 6:** Results of pairwise Fst analysis among clusters of Kersting’s groundnut accessions.

Global test				
Overall Weir and Cockerham's Fst-value^a^ = 0.62***				
Clusters	I	II	III	IV
**I (White seeded)**	-			
**II (Red seeded)**	0.82***	-		
**III (Black seeded)**	0.80***	0.54***	-	
**IV (White with black eye)**	0.92***	0.30***	0.76***	-

^a^ F-statistic measuring the degree of differentiation of the groups

*** indicates significance at p-value<0.001

However, the within cluster expected heterozygosity ranged from 0.01 to 0.09, revealing a low genetic diversity within clusters (cultivated landraces) of Kersting’s groundnut. The highest expected heterozygosity was obtained with Cluster 2 (He = 0.09) while Cluster 1 exhibited the lowest expected heterozygosity (He = 0.01). Clusters 3 and 4 showed an expected heterozygosity of 0.05 and 0.03 respectively.

Moreover, the analysis of phenotypic variance among clusters revealed high significant phenotypic differences between clusters for most morphological traits, including plant height, petiole length, leaflet length, 100 seed weight, seed length, seed width, seed thickness, days to 50% flowering, days to maturity, grain yield per plant and number of seeds per pod (Table 7). Cluster I was composed of late flowering (49.3±0.98 days) and late maturing (112.7±2.17 days) accessions with low grain yield per plant (4.34±1.89 g/plant). Clusters II and III were composed early flowering accessions (42.6±2.68 days for cluster II, and 41.5±2.31 days for cluster III), early maturing accessions (105.6±1.46 days for cluster II, 103.5±1.33 days for cluster III) with highest grain yield per plant (5.1±2.32 days for cluster II, 5.2±1.62 days for cluster III), highest seed size and highest 100 seed weight (12.49±2.16 g for cluster II, 13.39±1.25 g for cluster III) (Table 4). In contrast to cluster III, accessions of cluster II exhibited the highest values for leaf morphological traits. Cluster IV was consisted of earliest flowering (38.6±1.31 days) and early maturing (103.8±1.60 days) with low grain yield per plant (4.84±1.84 g/plant) and 100 seed weight (10.85±1.67 g) (Table 4).

**Table 7:** Phenotypic means and standard deviation of clusters of Kersting’s groundnut accessions and results of F-test of differences among clusters.

Variables	Cluster I	Cluster II	Cluster III	Cluster IV	F-value
	n = 217	n = 18	n = 40	n = 6	
**DIP**	19.68±1.68	20.59±1.72	20.05±1.65	19.73±0.56	1.90ns
**PLH**	14.28±1.26^a^	15.45±1.43^b^	14.33±1.16^a^	14.95±1.75^a^	4.53**
**LEL**	6.75±0.44^a^	7.03±0.50^a^^b^	6.97±0.41^b^	6.83±0.40^a^^b^	4.17**
**PEL**	7.56±0.67^a^	8.15±0.60^b^	7.41±0.62^a^	7.68±0.75^a^^b^	4.90**
**LEW**	4.23±0.30^a^	4.02±0.27^b^	4.37±0.41^c^	3.94±0.19^a^^b^	5.96***
**100SW**	10.49±1.19^a^	12.49±2.16^b^	13.39±1.25^c^	10.85±1.67^a^	66.60***
**SIL**	7.73±0.38^a^	8.29±0.36^b^	8.27±0.41^b^	7.70±0.50^a^	4.54***
**SWi**	5.35±0.25^a^	5.69±0.20^b^	5.79±0.24^b^	5.11±0.25^a^	44.19***
**STh**	4.11±0.17^a^	4.19±0.24^a^	4.14±0.18^a^	3.84±0.29^b^	4.48**
**DFF**	49.25±0.98^a^	42.56±2.68^b^	41.52±2.31^b^	38.57±1.31^c^	509.00***
**DTM**	112.7±2.17^a^	105.64±1.46^b^	103.47±1.33^c^	103.81±1.60^b^^c^	284.50***
**GRY**	4.34±1.89^a^	5.07±2.32^b^	5.21±1.62^b^	4.84±1.84^a^	2.98*
**NSP**	39.40±14.70	38.83±15.51	37.12±10.09	40.12±13.46	0.30ns
**NPP**	31.72±11.27	30.83±11.25	27.94±7.56	32.27±11.63	1.37ns
**NSPod**	1.23±0.07^a^	1.26±0.07^a^	1.31±0.07^b^	1.25±0.08^a^^b^	14.30***

DIP = diameter of plant (cm), PLH = plant height (cm), LEL = leaflet length (cm), LEW = leaflet width (cm), PEL = petiole length (cm), 100SW = 100 seed weight (g), SIL = seed length (mm), SWi = seed width (mm), STh = seed thickness (mm), DFF = days to 50% flowering (days), DTM = days to maturity (days), GRY = grain yield (g/plant), NSP = number of seeds per plant, NPP = number of pods per plant, NSPod = number of seeds per pod

a, b, c are used to separate means of clusters, values followed by the same superscript letter are statistically identical, ns = non-significant

*, **, *** indicate significance at p-values of 0.05, 0.01, and 0.001, respectively.

### Model-based population structure and phenotypic variation between subpopulations

**Fig 3:**
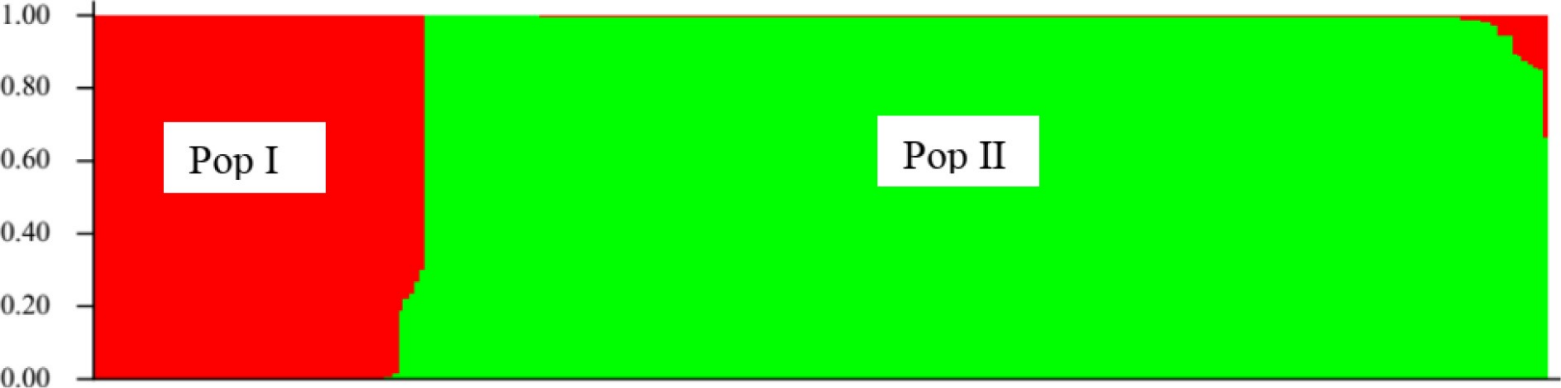
Barplot of populations sorted by Kinship matrix (Pop I = population I, Pop II = population II).

The admixture model-based clustering, using the 281 accessions, showed two distinct populations of Kersting’s groundnut accessions (Fig 3). Population I (Pop I) was composed of 64 accessions (22.78%) while population II (Pop II) consisted of 217 accessions (77.22%). Divergence rates of populations I and II from the hypothetical ancestral population built by the Bayesian clustering method, were estimated by mean Fst-values of 0.57 and 0.69, respectively. Therefore, populations I and II were highly differentiated from the hypothetical ancestral population. Moreover, the two populations were highly discriminated by agro-ecological origins of accessions and seed coat colours. About 87.5% of accessions of population I were collected in the Sudanian region while only 12.5% of them originated from the Guinean region. In contrast, all accessions of population II originated from the Sudano-Guinean (71.4%) and the Guinean regions (28.6%). In addition, population I included only white-seeded accessions while population II was composed of colourful accessions, e.g. red-seeded, black-seeded and white-seeded with black eye accessions. This reveals a high allelic differentiation between white-seeded and colourful accessions.

Results of Fst statistics depicting the degree of differentiation among accessions from different agro-ecological regions are shown in Table 8. High genetic differentiation was observed among regions with overall Weir and Cockerham's Fst-value of 0.37. Pairwise Fst-values varied from 0.07 to 0.59 (Table 8). The lowest Fst-value (0.07) was observed between the Guinean and the Sudano-Guinean regions. Relatively high Fst-value (0.25) was observed between the Guinean and the Sudanian regions. Moreover, the highest Fst-value (0.59) was detected between the Sudanian and the Sudano-Guinean agro-ecological regions.

**Table 8:** Results of pairwise Fst analysis among agro-ecological regions of Kersting’s groundnut accessions.

Global test			
Overall Weir and Cockerham's Fst-value = 0.37**			
Agro-ecological region	Guinean	Sudano-Guinean	Sudanian
**Guinean**	-		
**Sudano-Guinean**	0.07*	-	
**Sudanian**	0.25**	0.59***	-

^a^ F-statistic measuring the degree of differentiation of agro-ecological regions

*, **, *** indicates significance at p-value 0.05, 0 .01 and 0.001, respectively.

The two-sided Student test revealed high significant differences between both populations for the diameter of plant, leaflet length, 100 seed weight, seed length, seed width, days to 50% flowering, days to maturity, grain yield per plant and number of seeds per pod (Table 9). Contrary to population II, accessions of population I were early flowering (41.6±2.52 days), early maturing (104.1±1.67 days) and showed the highest 100 seed weight (12.98±1.69 g), seed length (8.24±0.42 mm), seed width (5.71±0.29 mm), grain yield per plant (5.15±1.82 g/plant) and number of seeds per pod (1.29±0.07) (Table 9).

**Table 9:** Phenotypic means and standard deviation of subpopulations of Kersting’s groundnut accessions and results of Student test of differences between subpopulations.

Variables	Population I	Population II	t-value
	n = 64	n = 217	
DIP	20.17±1.63	19.67±1.68	-2.09*
PLH	14.67±1.35	14.28±1.26	-2.00ns
LEL	6.97±0.43	6.75±0.44	-3.50***
PEL	7.63±0.69	7.55±0.67	-0.77ns
LEW	4.24±0.40	4.23±0.30	-0.26ns
100SW	12.98±1.69	10.49±1.19	-10.66***
SIL	8.24±0.42	7.73±0.38	-8.45***
SWi	5.71±0.29	5.34±0.25	-9.05***
STh	4.13±0.22	4.11±0.17	-0.88ns
DFF	41.60±2.52	49.25±0.98	22.94***
DTM	104.08±1.67	112.7±2.17	33.33***
GRY	5.15±1.82	4.34±1.89	-3.04**
NSP	37.79±11.80	39.4±14.70	0.89ns
NPP	29.02±8.92	31.71±11.27	1.97ns
NSPod	1.29±0.07	1.23±0.07	-5.57***

DIP = diameter of plant (cm), PLH = plant height (cm), LEL = leaflet length (cm), LEW = leaflet width (cm), PEL = petiole length (cm), 100SW = 100 seed weight (g), SIL = seed length (mm), SWi = seed width (mm), STh = seed thickness (mm), DFF = days to 50% flowering (days), DTM = days to maturity (days), GRY = grain yield (g/plant), NSP = number of seeds per plant, NPP = number of pods per plant, NSPod = number of seeds per pod

*, **, *** indicate significance at p-values of 0.05, 0.01, and 0.001, respectively.

### Marker-traits associations in Kersting’s groundnut

Based on the 113 SNP markers included in the GWAS analysis, the corrected Bonferroni threshold for significant marker-trait associations was p-value = 4.42 x 10^−4^. Significant SNP-traits associations in Kersting’s groundnut in specific sets of environments are shown in Table 10. Both the MLM and MLM-Q analyses revealed 10 SNP markers significantly associated with grain yield per plant and related traits across environments. Six of the marker-trait associations were repeated in at least the two sets of environments while the four other associations were environment-specific (Table 10). The analysis of Quantile-Quantile (QQ) plots showed good relationships between the expected and observed p-values for all studied traits (S1 Fig). The Marker M1 was significantly associated with 100 seeds weight at Sékou, Savè and for the overall environment and accounted for over 24% of the phenotypic variation. Markers M2 and M4 were respectively associated with days to 50% flowering and grain yield per plant at Sékou and for the overall environment (Table 10). Similarly, the markers M2 and M4 were respectively associated with days to maturity and the number of seeds per plant in all environments. Markers M5 and M6 were associated respectively with one hundred seeds weight and days to 50% flowering at Savè and for the overall environment. Marker M7 was significantly associated with the number of pods per plant at Savè and in the overall environment. Moreover, the marker M3 was discovered at Sékou in a significant association with days to maturity. In addition, the marker M7 was significantly associated with days to maturity and the number of seeds per plant at Savè. Other significant associations in the overall environment included markers M8, M9 and M10. The marker M8 was associated with days to 50% flowering and days to maturity while both markers M9 and M10 were associated with days to 50% flowering (Table 10). Markers M2, M6, M8, M9 and M10 were associated with days to 50% flowering with R^2^ values ranging from 10.6 to 25.4%. M2, M3 and M7 were associated with days to maturity. Grain yield per plant was correlated to the yield components of 100 seed weight, number of seeds per plant, number of seeds per pod, and number of pods per plant. Marker M4 was associated with grain yield per plant and number of seeds per pods. M1 and M5 were associated with 100 seed weight but not grain yield per plant and M7 was associated with number of seeds per plant but not grain yield per plant.

**Table 10:** GWAS results within and across environments for grain yield and related traits.

Env	Traits	Marker	Allele identity	Allele	p-value	-log10(p)	Add	SnpR^2^ (%)	MAF
**Sékou**	100SW	M1	100027985|F|0–60:A>G-60:A>G	G:G	2.50E-04	3.60	0.18	24.45	0.08
**Sékou**	DFF	M2	100075398|F|0–45:T>C-45:T>C	C:C	1.49E-04	3.83	0.09	25.38	0.07
**Sékou**	DTM	M3	100030821|F|0–34:A>C-34:A>C	C:C	1.28E-04	3.89	-0.02	23.57	0.22
**Sékou**	GRY	M4	100032049|F|0–6:G>T-6:G>T	T:T	8.53E-05	4.06	-24.20	8.71	0.08
**Savè**	100SW	M5	100031485|F|0–27:G>A-27:G>A	A:A	9.99E-05	4.00	0.07	11.46	0.15
**Savè**	100SW	M1	100027985|F|0–60:A>G-60:A>G	G:G	1.33E-04	3.87	0.33	25.55	0.08
**Savè**	DFF	M6	100048752|F|0–13:G>A-13:G>A	A:A	7.49E-07	6.13	-0.07	10.97	0.23
**Savè**	DTM	M7	100070037|F|0–60:G>A-60:G>A	A:A	2.27E-04	3.64	-0.02	24.60	0.24
**Savè**	NPP	M7	100070037|F|0–60:G>A-60:G>A	A:A	4.89E-06	5.31	-0.27	80.05	0.24
**Savè**	NSP	M7	100070037|F|0–60:G>A-60:G>A	A:A	3.67E-04	3.44	-0.25	4.23	0.24
**Overall**	100SW	M1	100027985|F|0–60:A>G-60:A>G	G:G	3.28E-04	3.48	4.60	24.23	0.08
**Overall**	100SW	M5	100031485|F|0–27:G>A-27:G>A	A:A	7.80E-05	4.11	-0.13	95.80	0.15
**Overall**	DFF	M8	100030725|F|0–48:G>T-48:G>T	T:T	6.48E-07	6.19	-1.11	10.81	0.08
**Overall**	DFF	M9	100031216|F|0–19:C>T-19:C>T	T:T	2.66E-04	3.58	2.69	24.36	0.06
**Overall**	DFF	M10	100031465|F|0–16:G>T-16:G>T	T:T	2.12E-04	3.67	-1.83	24.53	0.15
**Overall**	DFF	M6	100048752|F|0–13:G>A-13:G>A	A:A	1.99E-04	3.70	-3.92	24.58	0.23
**Overall**	DFF	M2	100075398|F|0–45:T>C-45:T>C	C:C	1.28E-07	6.89	12.64	10.59	0.07
**Overall**	DTM	M8	100030725|F|0–48:G>T-48:G>T	T:T	2.14E-04	3.67	1.16	26.26	0.08
**Overall**	DTM	M2	100075398|F|0–45:T>C-45:T>C	C:C	4.70E-05	4.33	13.71	61.41	0.07
**Overall**	GRY	M4	100032049|F|0–6:G>T-6:G>T	T:T	6.53E-05	4.19	-56.59	3.71	0.08
**Overall**	NPP	M7	100070037|F|0–60:G>A-60:G>A	A:A	3.81E-04	3.42	-31.27	4.12	0.24
**Overall**	NSP	M4	100032049|F|0–6:G>T-6:G>T	T:T	5.17E-05	4.29	-49.20	3.89	0.08

Env = environment, Add = additive effect, MAF = minor allele frequency, SnpR^2^ = proportion of phenotypic variation explained by marker

### Genomic selection models and accuracy in Kersting’s groundnut

The ridge regression analysis, including the 493 SNP markers, revealed moderate (0.42–0.44) to high (0.62–0.79) prediction accuracy for leaflet length, 100 seed weight, seed length, seed width, days to 50% flowering and days to maturity, using the stratified (involving accessions from all clusters) cross-validation sampling technique (Table 11). Moderate correlations were detected between observed and predicted values of leaflet length (0.44), seed length (0.43) and seed width (0.42). Strong correlations were detected between observed and predicted 100 seed weight (0.62), days to 50% flowering (0.79) and days to maturity (0.72). Low prediction accuracy was observed for the diameter of plant (0.17), plant height (0.15), petiole length (0.11), leaflet width (0.12), grain yield per plant (0.18), number of seeds per plant (0.20), number of pods per plant (0.18) and number of seeds per pod (0.16) (Table 11). The cross-validation approach including only accessions from cluster I (within cluster sampling) revealed low model accuracy (0.02 to 0.30) for all morphological traits (Table 11).

**Table 11:** Genomic selection models and prediction accuracy in Kersting’s groundnut using stratified and within cluster sampling techniques.

Variables	Genomic selection model	Accuracy of stratified sampling	Accuracy of within cluster sampling
**DIP**	19.95 + Xg_DIP_ + 0.41	0.17	0.17
**PLH**	14.68 + Xg_PLH_ + 1.18	0.15	0.12
**LEL**	6.87 + Xg_LEL_ + 0.34	0.44	0.14
**PEL**	38.62 + Xg_PEL_ + 0.60	0.11	0.19
**LEW**	4.17 + Xg_LEW_ + 0.24	0.12	0.21
**100SW**	10.94 + Xg_100SW_ + 3.55	0.62	0.02
**SIL**	8.17 + Xg_SIL_ + 0.78	0.43	0.03
**SWi**	5.50 + Xg_SWi_ + 0.63	0.42	0.16
**DFF**	43.65 + Xg_DFF_ + 5.08	0.79	0.06
**DTM**	108.11 + Xg_DTM_ 5.66	0.72	0.30
**GRY**	4.71 + Xg_GRY_ + 0.68	0.18	0.05
**NSP**	38.62 + Xg_NSP_ + 0.95	0.20	0.05
**NPP**	29.79 + Xg_NPP_ + 3.95	0.18	0.07
**NSPod**	1.27 + Xg_NSPod_ + 0.08	0.16	0.19

DIP = diameter of plant (cm), PLH = plant height (cm), LEL = leaflet length (cm), LEW = leaflet width (cm), PEL = petiole length (cm), 100SW = 100 seed weight (g), SIL = seed length (mm), SWi = seed width (mm), STh = seed thickness (mm), DFF = days to 50% flowering (days), DTM = days to maturity (days), GRY = grain yield (g/plant), NSP = number of seeds per plant, NPP = number of pods per plant, NSPod = number of seeds per pod, X = SNP markers, g = SNP markers effects.

## Discussion

### Single nucleotide polymorphism and genetic diversity among Kersting’s groundnut landraces

The discovery of good quality molecular markers is important to enhance the application of enabling biotechnologies for orphan crops improvement [6]. This study reports for the first time 493 SNP markers in Kersting’s groundnut, which were further quality assessed to obtain 113 high polymorphic and informative markers with MAF≥0.05 and a high reproducibility (0.99). Given the relative small number of SNP markers, Kersting’s groundnut is not as polymorphic as other self-pollinated species [61–63]. The average heterozygosity (He = 0.22) and total gene diversity (Ht = 0.43) across markers revealed a high genetic diversity within Kersting’s groundnut and a strong population structure. This finding reveals higher gene diversity than values reported by Pasquet *et al*. [20] on Kersting’s groundnut using biochemical markers, and Wang *et al*. [64], Ren *et al*. [65] on peanut (*Arachis hypogea* L.) based on single sequence repeat (SSR) markers. Moreover, our results revealed a low alignment (10.9%) of SNP markers to reference genomes of closely related species such as adzuki bean and mung bean in contrast to findings of Ho *et al*. [66] on bambara groundnut [*Vigna subterranean* (L.) Verdc.]. Consequently, whole genome sequencing is crucial in Kersting’s groundnut to make a reference genome available to increase the accuracy of SNPs calling and breeding prospects.

The results also showed the importance of SNP markers in revealing high genetic differentiation among Kersting’s groundnut accessions (Fst = 0.79). Very high genetic differentiation was observed among the four types of landraces included in this study, that is, white, red, black and white with black eye seeded accessions. Similar results were reported by Mohammed *et al*. [67] who observed genetic variation among five different Ghanaian accessions using 12 single sequence repeat (SSR) markers. These findings imply that cultivated landraces of Kersting’s groundnut encompass a high genetic differentiation in contrast to findings of Pasquet *et al*. [20] who used 19 enzymes (biochemical markers) on 20 accessions of Kersting’s groundnut. SNP markers are highly codominant, polymorphic and more appropriate to unveil the existing genetic diversity within a species as opposed to biochemical markers which are not abundant and reduce the resolution of the genetic diversity [25, 26]. On the other hand, the population structure analysis, including geographic origins of accessions, identified two subpopulations that were found to be highly structured, revealing the influence of geographic origins on the genetic diversity within Kersting’s groundnut. Large genetic differentiation was observed among accessions based on agro-ecological regions since the overall Fst = 0.37, which is greater than 0.25. Low genetic differentiation was detected between the Guinean and Sudano-Guinean regions. This might be because of the proximity of these regions and seed exchange among farmers. Kersting’s groundnut farmers in the Guinean and the Sudano-Guinean regions buy seeds on the markets [15]. However, great genetic differentiation was observed between the Sudanian and the two other agro-ecological regions. According to Akohoué *et al*. [15], farmers in the Sudanian region reused seeds from the previous harvest. The white with black eye seeded landrace was also reported to be specific to the Sudanian region. Further investigation in that region may reveal more diversity. The clear separation between early and late accessions as shown by both clustering and structure analyses could be explained by the high correlation between time to flowering and seed coat colour as reported by [68].

Moreover, the difference between the number of clusters revealed by the neighbour joining analysis and results of population structure could be attributed to limitations of the STRUCTURE software to adequately describe the structure of the population. Among other limitations, STRUCUTRE results are sensitive to sample size, number of populations, number of loci scored and the type of markers [53]. Despite these limitations, it was informative to present both perspectives so that readers appreciate the possible incongruence of results when using different computation approaches. Similar incongruence was reported by Al-Abdallat *et al*. [39] when the neighbour joining analysis revealed several subgroups of barley (*Hordeum vulgare* L.) accessions while STRUCTURE identified two distinct subpopulations.

### Broadening the genetic base within Kersting’s groundnut landraces

The improvement of Kersting’s groundnut requires the development of improved varieties for the most cultivated landraces, e.g. the white-seeded landrace. However, this study revealed a low genetic diversity within landraces, particularly the white-seeded landrace (He = 0.01) which is the most cultivated landrace due to the high economic value of its grains in most west African countries [15]. The low genetic diversity within landraces is likely due to the self-pollination mode of the species, the active geocarpic and chasmogamous nature of the flowers [20, 69]. Among other disadvantages, the geocarpy in Kersting’s groundnut limits seed or fruit dispersal, influences gene transfer and population genetic structure, and increases reproductive costs in the species [69]. Given the high phenotypic differences among clusters for grain yield and yield-related traits, the successful breeding of Kersting’s groundnut requires intensive cross pollinations among all landraces (e.g. white-seeded, red-seeded, black-seeded and white-seeded with black eye landraces) for a broader genetic diversity and improved gains from selection. Considering the influence of geographic origins on the distribution of landraces, the enhancement of the genetic diversity within Kersting’s groundnut requires also the introduction of new germplasm and crossing among genotypes from different production countries and regions. In addition, the available germplasm of Kersting’s groundnut could be enhanced through mutation breeding techniques, using chemical mutagenesis combined with the Targeted Induced Local Lesions in Genomes (TILLING). Mutation breeding has been successfully used to create genetic diversity and identify favourable mutants in many self-pollinated crops, including tomato (*Solanum lycopersicum* L.) [70] and soybean [*Glycine max* (L.) Merr.] [71].

### Marker-trait associations and genomic selection accuracy in Kersting’s groundnut

Phenotypic evaluation studies in Kersting’s groundnut showed great phenotypic variability among accessions [17, 72, 73]. From the results of this study, the broad sense heritability of most morphological traits was greater than 0.50, showing the presence of genetic variability among accessions across environments. Therefore, GWAS was performed to associate the phenotypic variation of yield and related traits with the observed molecular genetic diversity. A similar approach has been used on major legumes crops including cowpea [74] and peanut [75] to decipher the genetic basis of morphological traits in a set of environments. The GWAS analysis detected 10 markers significantly associated with grain yield and related traits. Six of the markers, including M1, M2, M4, M5, M6 and M7, were consistent across environments. Nevertheless, the other markers identified in this study were not clearly consistent across the two environments.

The inconsistency of GWAS results could be explained by the highly significant genotype by environment (GxE) interaction observed for most morphological traits included in this study, and different genetic mechanisms under drought conditions as reported by Al-Abdallat *et al*. [39], Varshney *et al*. [76] in barley (*Hordeum vulgare* L.). In this study, average rainfall recorded during field trials was lower than the water requirement of 500–900 mm/year of Kersting’s groundnut [14, 19]. In addition, dissecting the genetic basis governing complex traits using GWAS on a natural population in dry environments could be less informative compared with bi-parental and specialised mapping populations [76]. Conventional genome-wide association studies also perform poorly for rare variants that might be prominent, particularly for self-pollinated species [77]. The high R^2^ values (>26%) observed for some marker-trait associations suggests the presence of confounding phenotypic variation, revealing that including the three first principal components in the GWAS analysis did not adequately adjust for accessions clustering and population structure. This confounding effect between some of the markers and phenotypic variation arises from the high significant differences in the phenotypic variance among Kersting’s groundnut clusters and subpopulations [78].

Despite these limitations, the GWAS provided a first insight into the genetic basis of farmers’ preferred traits in Kersting’s groundnut. Further investigation on the whole genome assembly is required for a clear identification of chromosome position of single nucleotide polymorphisms in the species. In addition, given the low genetic base within landraces, the development of specialised mapping populations like Multi-parent Advanced Generation Inter-cross (MAGIC) populations could be relevant for the accurate identification and mapping of quantitative traits loci (QTLs) in Kersting’s groundnut as reported in many self-pollinated crops including rice (*Oryza sativa* L.) [79] and cowpea [80]. In contrast to bi-parental populations (e.g. F_2_ and backcross populations, recombinant inbred lines, near isogenic lines and double haploids), MAGIC populations increase the recombination rate and genetic diversity, and reduces the extent of linkage disequilibrium (LD), giving the opportunity to detect more QTLs with a higher precision [81, 82]. Cultivated landraces could serve as founder lines that could be mixed through inter-crossing to form a broader genetic base.

In addition to the GWAS, genomic selection models, using the stratified sampling technique, revealed moderate to high prediction accuracies for leaflet length, seed dimension traits, days to 50% flowering and days to maturity. The high prediction accuracy revealed by the stratified sampling technique could be explained by the existence of high relatedness among accessions. On the other hand, the within cluster sampling technique revealed very low to moderate prediction accuracies for all traits. This finding implies that the application of genomic selection for the improvement of the crop requires the development bi-parental and specialised mapping populations. The utilisation of these populations having low population structure could maximize accuracy and selection gains and accelerate the deployment of improved Kersting’s groundnut varieties with farmers’ preferred traits.

## Conclusion

In this study, the genetic diversity, marker-trait association patterns and possibility for accurate genomic selection within a west African collection of Kersting’s groundnut are described. In total, 493 SNP markers were discovered, of which 113 showed a minor allele frequency ≥0.05. High mean heterozygosity and total gene diversity were observed within the species. The analysis of genetic diversity revealed four clusters of accessions significantly discriminated by seed coat colours namely the white seeded, red seeded, black seeded and the white with black eye seeded accessions. However, a low genetic diversity was observed within clusters. The population structure revealed great genetic differentiation across agro-ecological regions of accessions. Further, the GWAS analysis detected 10 markers associated with yield and related traits. Six of the markers showed clear consistency across environments while the remainder were environment-specific. The genomic selection analysis revealed moderate accuracy for leaflet length and seed dimension traits, and high prediction accuracies for 100 seed weight, days to 50% flowering and days to maturity. SNP markers identified in this study could be useful for marker-assisted selection in Kersting’s groundnut breeding programmes. Further investigations are required regarding the creation of broader genetic diversity within landraces, development of specialized mapping populations and the assembly of the genome of Kersting’s groundnut to enable appropriate association mapping with clear chromosome positions.

## Supporting information

S1 Table Characteristics of the 113 SNP markers with minor allele frequencies (MAF) > 0.05 in 281 accessions of Kersting's groundnut. (XLSX)

S1 Fig Quantile-Quantile (QQ) plots of the mixed linear model including the Kinship matrix (MLM-Q). (DOCX)

S1 Data (XLSX)
