# Supplementary material for: Genetic diversity, SNP-trait associations and genomic selection accuracy in a west African collection of Kersting’s groundnut [Macrotyloma geocarpum(Harms) Maréchal & Baudet]
Source: PLoS One. 2020 Jun 30;15(6):e0234769. doi: 10.1371/journal.pone.0234769 (PMC7326195; doi:10.1371/journal.pone.0234769)
Supplement: S1 Fig — (DOCX) [file pone.0234769.s002.docx]

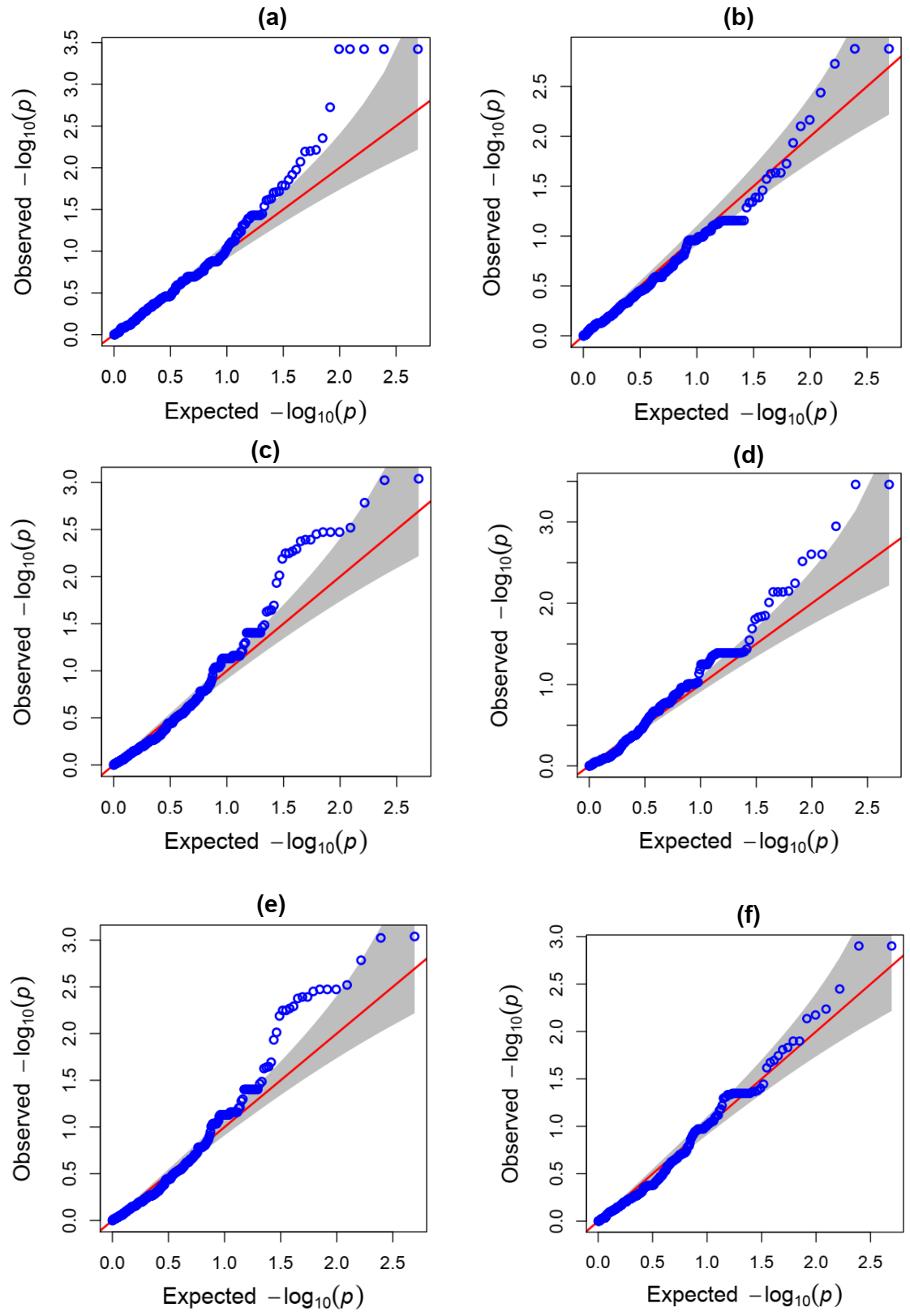


S1 Fig: Quantile-Quantile (QQ) plots of the Mixed Linear Model including the Kinship matrix (MLM-Q); (a)= 100 seeds weight (100SW), (b)= grain yield per plant (GRY), (c)= days to 50% flowering (DFF), (d)= days to maturity (DTM), (e)= number of seeds per plant (NSP), (f)= number of pods per plant (NPP)
